# Combination regimens with PD-1/PD-L1 immune checkpoint inhibitors for gastrointestinal malignancies

**DOI:** 10.1186/s13045-019-0730-9

**Published:** 2019-04-24

**Authors:** Dongxu Wang, Jianzhen Lin, Xu Yang, Junyu Long, Yi Bai, Xiaobo Yang, Yilei Mao, Xinting Sang, Samuel Seery, Haitao Zhao

**Affiliations:** 1 Department of Liver Surgery, Peking Union Medical College Hospital, Chinese Academy of Medical Sciences and Peking Union Medical College, Beijing, China; 2 Department of Humanities, Peking Union Medical College, Chinese Academy of Medical Sciences and Peking Union Medical College, Beijing, China

**Keywords:** Gastrointestinal malignancies, PD-1/PD-L1 blockade, Immune checkpoint inhibitor, Combination immunotherapy, Clinical application, Rationale, Clinical trial

## Abstract

Gastrointestinal (GI) malignant neoplasms have a high global incidence and treatment prospects for patients with advanced GI tumors are dismal. PD-1/PD-L1 inhibitors emerged as a frontline treatment for several types of cancer. However, the shortcomings of PD-1/PD-L1 inhibitors have been observed, including low objective response rates and acquired tumor resistance, especially in patients receiving PD-1/PD-L1 inhibitors as a single treatment. Accumulating evidence from clinical trials increasingly suggests that combined immunotherapies enhance therapeutic responses in patients with malignances, especially for GI tumors which have a complex matrix, and significant molecular and immunological differences. Preclinical and clinical studies suggest there are advantages to combined immunological regimens, which represents the next logical step in this field, although further research is necessary. This literature review explores the current limitations of monotherapies, before critically discussing the rationale behind combination regimens. Then, we provide a summary of the clinical applications for gastrointestinal cancers.

## Background

Gastrointestinal (GI) neoplasms threaten human health and account for approximately 35% of all cancer-related mortalities among common malignancies [1]. Typically, patients are diagnosed accidentally with latent, unspecific symptoms reducing the already limited number of possible interventions. Surgical resection can be curative; however, the majority of patients are diagnosed in the advanced stages of this condition, therefore the opportunity for a radical cure is lost. The prevalence and impact of this insidious disease as well as limited treatment options necessitates the systematic search for innovative evidence-based treatments.

Advances in our understanding of immune-system/tumor interactions have led researchers to uncover new diagnostic pathways which may result in earlier identification. Also, several immunotherapies for the treatment of GI tumors have recently emerged. Among these new interventions, immune checkpoint inhibitor therapies are perhaps the most promising strategy [2]. Indeed, the findings from many clinical trials suggest that immunological checkpoint blockade therapies may be effective for various types of tumor, with durable responses and manageable toxicity, regardless of pathologic grade [3]. For those with GI tumors, blocking programmed cell death protein-1 (PD-1/CD279) or the ligand PD-L1 is also effective in approximately 20–40% patients. Due to such outcomes and with this moderate success, PD-1/PD-L1 blockades have been approved by the FDA for advanced colorectal, gastric, and liver cancers.

In contrast to other tumors such as lung cancer and breast cancer, GI tumors have mesenchymal traits which hinder the infiltration of immune cells thereby crippling the antitumor response [4]. Likewise, the immunotherapeutic effects upon digestive tract tumors vary substantially which is perhaps due to different molecular and immunological characteristics. As such, several researchers have called for GI tumors to be reclassified based upon molecular type rather than around anatomical systems and histological features only [5]. Despite this call for change, high mortality rates associated with these malignancies continues to drive clinical research in this field. Several phase I–III trials focusing on immunotherapies for GI tumors have found what can only be described as unsatisfactory objective response rates (ORR), ranging between 10 and 25% [6]. In addition, problems such as drug resistance and the side effects of anti-PD-1/PD-L1 treatments remain challenging [7]. So, while this growing body of evidence suggests that target-driven treatment strategies are essential, there is a paucity of research from which to design new interventions.

Presently, the logical next step appears to be combining immunotherapies with antitumor drugs and some progress has been made in preclinical and clinical studies which suggest that combined immunotherapies may increase benefit. However, this is a relatively new field of study hence effort should be made to embed research systematicity using secondary literature. As such, this study focuses on reviewing the current limitations of immune checkpoint blockade monotherapies and to critically discuss the rationale behind combination strategies based on the PD-1/PD-L1 blockade. The aim is to provide researchers and practitioners with a summary of the clinical applications of combination therapies for patients with upper and lower GI tumors and to explore the arguments around combination immunotherapies.

## PD-1/PD-L1 pathway blockade: current limitations in clinical treatment

The immune checkpoint pathway composed of PD-1/CD279 and the related ligand PD-L1 evade immune surveillance by upregulating the expression in tumor cells during the progress of T cell-mediated immune killing. Substantial evidence from preclinical models indicates that blocking PD-1/PD-L1 interactions can enhance immune normalization and reinforce anticancer responses [8, 9]. As early as 2003, Chen et al. found that using the B7 homolog 1 (B7-H1) blocking antibody combined with T cell transfusion cured approximately 60% of the 24 mice with squamous cell carcinomas in the head and neck. Without the transfusion of T cells, only one of five mice treated with B7-H1 blockade had prolonged survival; however, this was not considered a statistically significant improvement compared with the control group [10].

In 2012, a phase I clinical trial investigating the efficacy of pembrolizumab for patients with advanced tumors found that the objective response rate (ORR) for patients with advanced non-small cell lung cancer (NSCLC), malignant melanoma, and advanced renal carcinoma was 18%, 28%, and 27%, respectively, and the adverse event profile does not appear to preclude its use [11]. Similarly, a longitudinal study focusing on pretreated advanced NSCLC, involving 129 patients found a 16% five-year survival rate. While this study contained a larger number of participants which adds precision, pretreatments were not standardized. Nevertheless, this study suggests that PD-1 blockading may prolong therapeutic durability [12]. This evidence of antitumor activation and the antibodies targeting the capabilities of PD-1/PD-L1 convinced the FDA to officially approve five inhibitors. The preliminary indications were that these inhibitors could be administered for several different types of tumor, including microsatellite instability-high (MSI-H) solid tumors.

The main advantages of PD-1/PD-L1 inhibitors are effect persistence (i.e., durability) and the broad-spectrum effects of these agents. However, the noticeable deficiency of PD-1/PD-L1 blockades is *inconsistency* across a homogeneous study population with similar tumor characteristics [13]. The exception to this can be observed in tumors with specific genetic changes, such as MSI-H, deficient mismatch repair (dMMR), and high tumor mutational burden (TMB). A review of the status and perspectives of translational biomarkers found the ORR is only 15–25% for unscreened solid tumors and even lower for some tumors, such as colorectal and pancreatic cancer [14] which suggests the causal factor for this relatively low response rate might be attributed to tumor heterogeneity, genetic variation among individuals, and perhaps structural differences between blockades [15]. Although, studies have also found that the development and evolution within a tumor itself can lead to a decreased efficacy of the PD-1 blockade. This may be due to genetic alterations within DNA encoding immunogenic signaling pathway proteins, a lack of sufficient mutation-associated neoantigens (MANAs) in the presence of an immunosuppressive tumor microenvironment, and/or the unmasking of immunogenicity by immune checkpoint inhibitors (ICPIs) to induce an enhanced antitumor response [16].

As well as increasing antitumour activity, PD-1/PD-L1 blockade treatments may also cause certain inflammatory side effects in some patients which are referred to as immune-related adverse events (irAEs) [17, 18]. Essentially, these immunotherapies unbalance the immune system, generating dysimmune toxicities which potentially effect any tissue. However, a systematic review of the side effects of the PD-1/PD-L1 blockade suggests irAEs can be widespread but are more likely to involve the GI tract, endocrine glands, and skin [19–21]. Compared to the side effects of chemotherapy, immunotherapeutic side effects appear more diverse, random, and differential but primarily organ-based manifestations [17]. Some studies indicate that these irAEs may be closely related to the expression and distribution of PD-L1 and PD-L2 [22–24] which suggests while irAEs may be heterogeneous in nature, they may be tolerable and most associated side effects are treatable. However, there are potentially serious adverse reactions, such as myocarditis which can cause death. A substantial increase in the number of deaths associated with immune checkpoint inhibitors has been observed, although this may be attributed to increased use and raised awareness of this clinical entity [25]. Conversely, some irAE studies have found improved immune responses in patients which suggest that these might also be used to predict treatment efficacy [26].

The efficacy of PD-1/PD-L1 blockades can be lasting for some patients, although tumor development remains a constant threat even under continuous therapy [27]. In a screening evaluation of PD-1 for the treatment of malignant melanoma, 48 cases were found to have significantly reduced tumor size or stable progression. However, in approximately half of those participants, tumors initially shrank before increasing in size directly after receiving this intervention [28]. This suggests that this treatment may have had little or no effect overall due to immunotherapeutic resistance. At present, the possible mechanisms of acquired immunotherapy resistance appear to include loss-of-function mutations in beta-2-microglobulin (B2M) and Janus kinases (JAK1 and JAK2) [29].

A study of two fully immunocompetent mouse models focusing on lung adenocarcinoma indicate that the T cell immunoglobulin mucin-3 (TIM-3) was upregulated in tumors resistant to PD-1 blockade, and a survival advantage was found with the addition of a TIM-3 blocking antibody following failure of the PD-1 blockade. This suggests that there may be a targetable biomarker associated with adaptive resistance to PD-1 blockades [30]. Early clinical investigations have also found some patients with complete remission after treatment with PD-1/PD-L1 blockades, relapse. Although, data related to this phenomenon is limited, it does suggest a lack of therapeutic durability in humans which is supported by basic medical evidence.

Adding to the aforementioned side effects and drug resistance after immunotherapy, studies indicate that a small number of patients on PD-1 blockades will experience hyper-progression [31–33]. The Ferrara study, which included 242 patients, found that tumor growth rates increased by more than 50% in 16% of patients (*n* = 40) after receiving the PD-1 antibody. This finding meets the criteria for hyper-progression; however, this study lacked a control group and determining tumor progression causality was not possible [34]. To explore this phenomenon in more detail, Singavi et al. conducted an analysis of somatic alterations looking into the biomarkers for hyper-progression and found that copy number alterations in murine double minute 2/4 (MDM2/MDM4), the epidermal growth factor receptor (EGFR), and several genes located on 11q13 are associated with hyper-progression. The role of these somatic alterations as putative predictive biomarkers for hyper-progression requires further investigation with larger samples [35].

Identifying biomarkers is crucial as these might support both treatment efficacy and AE predictions in patients receiving immunological checkpoint therapy [36]. Biomarkers such as dMMR and MSI, TMB and blood TMB, HLA diversity and PD-L1 expression have been explored. While stable predictors are not, presently available, different regions of the body develop different types of tumor, therefore antibodies used for detecting PD-L1 expression may be highly specific to one region while insensitive to level of expression, and vice versa. Furthermore, the activation effect of subsequent treatments is likely to change PD-L1 expression [37], a factor which is currently adopted in clinical trials to predict immunological efficacy [38]. While TMB, dMMR, and MSI positively correlate with the efficacy of PD-1, they are not widely used due to the limitations of these detection techniques [39]. In summary, our knowledge of these biomarkers is far from complete therefore cannot be used as guidelines for precision immunotherapy. Alternative predictive markers are currently in the early exploratory phase [40, 41].

## Combination immunotherapy: future steps for effective immunotherapy

### Rationale behind combined immunotherapies based on PD-1/L1 blockade

The limitations of monotherapy with PD-1/PD-L1 blockades and the lack of promising alternatives has made it necessary to seek combination treatment methods which can activate antitumor immunity and enhance treatment efficacy. Studies on the interactions between the immune system and tumors indicate the cancer-immunity cycle primarily involves the following steps. Firstly, tumor-antigens are released to be processed by anti-presenting cells (APCs) which migrate to lymphoid organs. Then, T cells are activated and fine-tuned through co-stimulation and co-inhibitory signals which regulate naive tumor-specific T cells, encouraging them toward the tumor tissues and to become effector T cells in lymphoid organs. The regression of tumor-specific effector T cells which occurs from lymphoid organs into the peripheral blood is known as adoptive T cell transfer and trafficking. The final stage occurs when cytotoxic T cells attack tumor cells leading to tumor lysis as well as encouraging tumor-specific memory T cells [9, 42, 43]. From activation to cytotoxicity, T cell-led cellular immune regulation mainly progress through the final three steps described [43, 44].

Any abnormality in processing T cell immune clearance can lead to a decrease or even the disappearance of antitumor effects. Therefore, tumor tissues can escape a T cell attack through three regulatory mechanisms. There is the adaptive immune system and the natural immune systems inhibition of cell recruitment, such as the recruitment of regulatory T cells (Tregs) and myeloid-derived suppressor cells (MDSCs). Tumors can also escape variant selection and tumor-associated antigen expression loss, as well as co-stimulatory molecule downregulation and immunosuppressive factor secretion [45]. Based upon this current understanding, a hypothesis emerged that antitumor drugs which potentially enhance T cell immunity and interfere with tumor immune responses can be combined with a PD-1 blockade thereby exerting a synergistic antitumor effect. However, caution must be given because this is a generalized theory which may only apply to specific types of carcinoma.

GI neoplasms have a complex matrix microenvironment which includes a variety of cell types, extracellular matrices, and metabolic mediators [4, 46]. Each of these components can become obstacles for cytotoxic T lymphocytes (CTLs) which may enable tumor cells to evade attack by antitumor drugs. Different types of tumor cells have different levels of immunogenicity, related to cell surface antigens which activate immune cells. This variability creates different levels of tumor resistance and varying immune responses. As such, tumors can be categorized into different immunity-related phenotypes (i.e., immune-desert phenotype, immune-excluded phenotype or immune inflammatory phenotype) [47]. Within the immune desert phenotype, the tumor micro environment lacks immune-effector T cells because immunogenicity functions are inhibited. The result of which is ineffective T cell priming or activation. Although, sufficient effector T cells exist near the tumor in the immune-excluded phenotype, T cells appear to be blocked between the stroma and the tumor parenchyma. Therefore, T cells cannot penetrate to attack tumors. Both immune-desert and immune-excluded phenotype tumors are considered cold tumors, which are associated with low responsiveness [48].

Unlike immune-desert or immune-excluded phenotypes, immune inflammatory phenotypes are considered hot tumors which suggest they may be highly sensitive and responsive to treatment with a PD-1/PD-L1 blockade. One of the purposes of combination immunotherapies is to convert cold tumors into hot tumors by altering the tumor micro environment, thereby enhancing immune efficiency [48, 49]. Generally, tumors contain a large number of CD4/CD8^+^ T cells, APCs, and monocytes [47]. As such, combing PD-1/PD-L1 blockade therapies can be further rationalized because it is hoped this will enhance the presentation of tumor antigens, including the antigen-presenting function of antigen-presenting cells. Combining PD-1/PD-L1 may also enhance immune auxiliary functions thereby increasing the infiltration of T cells and the activity of CTLs in tumor tissues. Also, combination therapies may enhance tumor cell immunogenicity while reducing the efficacy of immunosuppressive molecules such as indoleamine 2,3-dioxygenase (IDO), C-X-C Motif chemokine receptor 2 (CXCR2), lymphocyte-activation gene 3 (LAG-3), phosphoinositide 3-kinase (P13K), for example [48, 50].

**Fig. 1:**
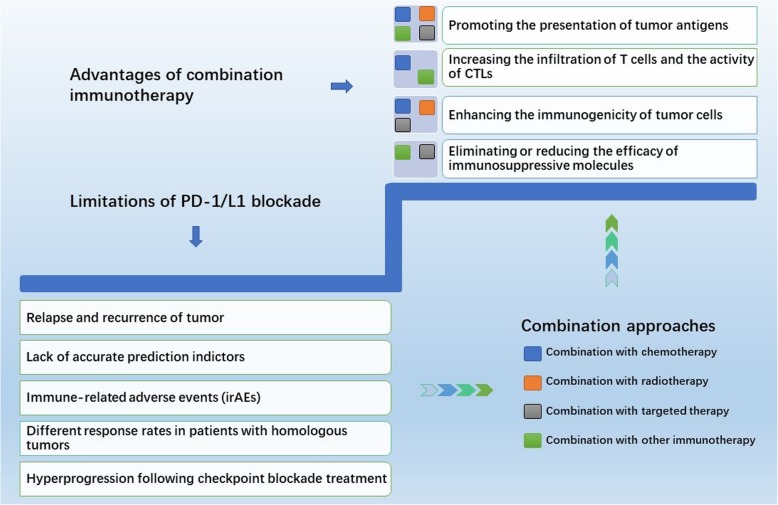
Limitations of PD-1/L1 blockade monotherapy and advantages of combination immunotherapy. There are currently many limitations of single-drug therapy with PD-1 inhibitors, including the five aspects shown above, but combined immunotherapy may help to solve some of the limitations of single-drug therapy. Specific combination immunotherapy strategies include combined radiotherapy, chemotherapy, targeted therapy, and another related immunotherapy

Traditional treatment methods, including combined radiotherapy with chemical interventions, have had limited success but can kill tumor cells which may lead to the release of tumor-specific antigens, thereby initiating immune clearance [51]. Targeted therapies focusing on tumor-specific gene mutation show promise and therefore are likely candidates for further investigation. In addition, evidence suggests PD-1/PD-L1 blockades can be combined directly with other immune checkpoint inhibitors (ICPIs), including some immunosuppressive small molecule blockades, having compatible, and theoretically complementary modalities. This emergent approach is commonly referred to as double immunotherapy and the mechanisms of multiple combined treatments are summarized below in Fig. 1.

The purpose of exploring combined interventions is to combine specific antitumor modalities to enhance therapeutic effects. However, this must be an evidence-based investigation to reduce both risk and harm. In contrast to immune monotherapy, several studies have shown that combined immunological regimens increase the incidences of all grade irAEs, especially for double immunotherapy [52]. The severity of adverse events in combination therapies also differs from monotherapies. For example, a study of nivolumab combined with ipilimumab compare to monotherapy for untreated melanoma found grade 3 or 4 irAEs occur in 55% of those in the combination cohort, while in the nivolumab cohort and ipilimumab cohort this was only 16.3% and 27.3%, respectively [53]. Of note, in contrast to the mechanisms of traditional tumor agents which target the tumor in situ, immunotherapies exert an antitumor effect through the activation of the immune system, creating different adverse events profiles. Under these circumstances, immunotherapies combined with traditional antitumor agents may result in a more complex set of adverse events [54, 55]. Therefore, combined regimens for immunotherapy present specific challenges that must be considered with respect to the evaluation of treatment related adverse events.

### Approaches and mechanisms for combining PD-1/PD-L1 with antitumor therapies

#### PD-1/PD-L1 blockades combined with other T cell checkpoint inhibitors

There are two critical steps of T cell activation which play an essential role in immune homeostasis. These steps involve multiple immune checkpoint pathways within the cancer-immunity cycle. Research suggests that several ICPIs may enhance the activity of cytotoxic T cells by antagonizing regulatory pathways which inhibit T cell functions [56]. Similarities and differences in checkpoint pathway mechanisms may be the reason single checkpoint inhibitors do not hold the desired antitumor effect. Combined immunotherapy targeting different immune checkpoints may then increase response rates.

Many immunological checkpoint combinations have been administered both in research and practice, including PD-1 combined with co-inhibitory factors, such as LAG-3, CTLA-4, and TIM-3. Several co-stimulatory factors such as tumor necrosis factor receptor super family member 4 (TNFRSF4), glucocorticoid-induced TNFR family related gene (GITR), and CD137 have also been investigated [57, 58]. PD-1/PD-L1 combined with CTLA-4 is currently one of the most commonly used immunological checkpoint combinations, and has been approved by the FDA for use in advanced renal carcinoma and melanoma. The mechanisms by which these agents affect immune function are subtly different and so necessary research is ongoing.

Presently, research indicates CTLA4 blockades act within lymph nodes, whereas PD-1/PD-L1 blockades act primarily in tumor tissues [59]. Basic research has demonstrated that after CD8^+^ T cells are removed, inhibiting the PD-1/PD-L1 pathways which fail to initiate tumor killing effects. On the other hand, CTLA-4 blockades also inhibit the B7-CTLA-4 pathway, which can initiate CD8^+^ T cell proliferation in lymph nodes and increase the infiltration of CTLs into tumor tissues [60]. Additionally, CTLA-4 antagonists may impede tumor inhibition capabilities of Treg cells. Therefore, in tumors with less T cell infiltration, this combination may have complementary action and clinical research supports this theory, indicating that this combination yields a higher level of treatment efficacy than either agent administered independently [60].

In one study of patients suffering advanced melanoma, the median overall survival (mOS) was surpassed with nivolumab and ipilimumab combined at the 36-month follow up point. Administered separately, the nivolumab and ipilimumab groups resulted in a 37.6 and 19.9 month mOS, respectively [61], indicating this combination does increase longevity for those suffering advanced melanoma. Similarly, in patients with advanced renal cell carcinoma, a study of a combination of first-line targeted drugs revealed a 42% ORR with nivolumab plus ipilimumab compared to 27% with sunitinib. This study found prolonged longevity where the mOS surpassed the 26 month baseline established with a sunitinib monotherapy [62]. Unfortunately, patients with negative PD-L1 expression are generally considered less likely to respond well to anti-PD-1 monotherapies, therefore may benefit from a combination.

Interestingly, the CheckMate-227 study which compared chemotherapy alone with double immunotherapy found that double immunotherapy can improve mPFS as well as ORR in patients suffering lung cancer, irrespective of PD-L1 expression. Overall, there was a 45.3% ORR with a corresponding 7.2 mPFS in those whom received nivolumab plus ipilimumab. This finding was in stark contrast to 26.9% mPFS and 5.5 month mOS found in those whom had received chemotherapy alone [63]. While these initial findings show promise, supporting evidence with which to generate systematic reviews or meta-analyses focusing on this field is sparse. Having said that, this evidence should be taken indicatively and should drive further research in this area.

#### PD-1/PD-L1 blockades combined with tumor immunotherapeutic small molecules

T cell chemokines, cell chemokine ligand 5 (CCL5), and C-X-C motif chemokine 10 (CXCL10) are associated with a better responses to immunotherapy [64]. The use of small molecular bioeffectors, such as histone deacetylase (HDAC) inhibitors which enhance the expression of T cell chemokines, may augment response rates to PD-1 blocking immunotherapy [65]. These small molecules can be classified into enzyme inhibitors (i.e., IDO and ARG1), chemokines and their receptors (i.e., the CXCR family), antigen-activated immune response classes (TLRs), signal transduction classes (PI3K-γ and BRAF), metabolites, cytokines, and other classes (e.g., COX2) [66]. Most small molecules develop during the preclinical stage; however, some small molecules enter the clinical stage, especially enzyme inhibitors and signal transduction factors. The guiding principle of the synergistic effect for combining small molecule drugs with ICPIs is that this may enhance tumor immunogenicity, which may in turn may enhance the efficacy of immuno-oncological (IO) treatments.

For instance, Indoleamine 2,3-dioxygenase 1 (IDO-1) which catalyzes the decomposition of tryptophan into kynurenic acid, may prevent CTLs from attacking cancer cells while upregulating Treg immunosuppression [67]. Evidence however remains conflicting, with early phase clinical trials suggesting that approximately 58% of melanoma patients (*n* = 19) receiving IDO inhibitors combined with PD-1 inhibitors achieved a complete response (CR) of 26% and a 32% partial response (PR) [68]. Likewise, in the ECHO-204 phase I/II study which combined an IDO inhibitor with nivolumab also yielded promising results in patients with melanomas with ORR and CR rates of 63% and 5%, respectively. The overall effect size was both enhanced and considered significant when compared with pharmacological interventions alone [69]. However, a recent phase III study suggests that perhaps this effect does not transpose and combining IDO inhibitors with pembrolizumab did not result in a significant, longer PFS when compared with placebo plus pembrolizumab (median 4.7 versus 4.9 months). The PFS rate at 12 months was 37% in both groups which suggests that there is no improvement. Unfortunately, combining these interventions also manifested in an increased number of side effects compared with PD-1 inhibitors alone [70]. Therefore, a full understanding of the mechanisms of small molecule drugs combined with ICPIs garnered through basic research and phase I/II trials is necessary before large phase III trials are commenced in this area.

#### PD-1 blockades combined with targeted therapy

Increasing attention is being given to targeted therapies because the identification of actionable oncogenic driver alterations has improved and we are gaining a deeper understanding of the microenvironments in which tumor develop. Monoclonal antibodies (McAbs) which target tumors mainly include drugs that target tumor-driving genes, inhibit protein kinase complexes by targeting the fusion mutation of EGFR, ALK, etc., or drugs which target angiogenesis (e.g., axitinib or sorafenib). Currently, the efficacy of targeted drugs is limited due to the development of acquired resistance initiated by different molecular mechanisms. However, this can be *partially* offset considering the durability of PD-1/PD-L1 inhibitors, which may exert a synergistic antitumor effect.

Neoantigens released through the lethal effects of targeted agents may actually strengthen the antitumor immune response [71]. In a melanoma mouse model, dabrafenib significantly increased the infiltration of CD8^+^ T cells, and trametinib in BRAF wild-type tumor cells appears to upregulate human leukocyte antigen (HLA) molecule expression while downregulating certain immunosuppressive factors such as PD-L1, IL1, IL8, CD73, and vascular endothelial growth factor A (VEGFA) [72]. Anti-angiogenesis drugs may normalize abnormal tumor blood vessels, thereby increasing the infiltration of immunocytes and exerting the anticipated synergistic antitumor effects of immuno-targeted therapy [73]. In addition, antiangiogenic treatment may ameliorate tumor hypoxia and transform the immunosuppressive tumor microenvironment into an immune-enhanced tumor microenvironment [74, 75], although clinical studies are required.

Thus far, a number of clinical trials have investigated PD-1 blockade combined with antiangiogenic drugs, including combinations with lenvatinib, cabozantinib, bevacizumab, and axitinib. The results of PD-1 blockade combined with lenvatinib suggest that there may be a benefit for patient suffering advanced renal carcinoma (63% ORR). However, this was a relatively small study (*n* = 30), therefore findings can be only tentatively generalized. In a similar study conducted involving 23 patients suffering endometrial carcinoma, researchers found a 50% ORR [76, 77] which might be considered promising, although not enough is known about the impact of demographic differences or lifestyles. Therefore, while promising, these studies should only be used to initiate larger studies, designed with more comprehensive data collection methods.

Taken together, these studies provide a small and incomplete evidence base for combining targeted drugs with ICPIs. At present, not enough is known about appropriate doses, time sequencing, or individuals which may improve patient prognosis. So, while the FDA has reported this combination as a “breakthrough” in the treatment of advanced renal cell carcinoma, caution must be given. Further, large-scale studies are required before such broad generalizations are presented publicly. Having said that, this is an area which does show promise and is the foundation of an emerging evidence base which should incorporate a focus on dose optimization, sequencing treatments, and demographic differences in order to maximum individual benefit.

#### PD-1 blockades combined with radiotherapy

There is a dual effect of radiotherapy on the immune system. On the one hand, radiotherapy inhibits immunity and promotes tumorigenesis. On the other hand, radiotherapy promotes tumor immunogenicity and apoptosis which enhances CD8 T cell tumor-infiltration while stimulating a systemic immune response [78]. Preclinical studies have shown that localized radiotherapy can promote the release of tumor-associated antigens, recruiting immune cells and change the tumor microenvironment which in turn promotes the antitumor immune response [79]. Adding a PD-1 inhibitor after radiotherapy has been administered and might manifest in a prolonged immune memory as has been observed in situ tumor vaccines [80]. Therefore, the role of radiotherapy as a treatment is evolving into perhaps a more powerful adjuvant for immunotherapy.

Radiotherapy can reduce MDSCs developing within the tumor microenvironment, producing new tumor antigens and potentially enhancing antigen presentation. Radiotherapy functions by destroying the tumor matrix and for a short period many antigens are released. These antigens are captured by dendritic cells and presented to T lymphocytes in order to produce lymphoid factors, which act on primary tumor cells. Consequently, localized radiotherapy may have a abscopal effect in various areas [81]. PD-1 blockades amplify these abscopal effects, and radiotherapy increases the expression of PD-L1 in tumor cells [82] which suggests intervention compatibility. Therefore, early evidence around the radiotherapy with PD-1 blockade combination can also be considered promising.

Animal studies involving mice have shown that the median survival time of those receiving radiation plus a PD-1 blockade was 53 days which is twice that of the single-drug immunotherapy group [83]. In addition to these theoretical points, the PACIFIC study focused on patients with locally advanced lung cancer who had been treated with durvalumab for 1 year after concurrent radiotherapy and chemotherapy. The results suggest that survival can be substantially prolonged by 16.8 months when compared with placebo which was only 5.6 months [84]. Similarly, the results of a prospective phase I clinical trial involving patients with metastatic solid tumors suggest that stereotactic radiotherapy combined with a PD-1 inhibitor results in a 44% ORR, with an mOS was 9.6 months and acceptable levels of toxicity [85].

Evidence from an increasing number of preclinical studies help rationalize and support combining radiotherapy with PD-1 blockades. However, there are only a few clinical studies focusing on this approach and most randomized clinical trials (RCTs) have been conducted with patients in the early stages of carcinoma development when responses may differ. Furthermore, knowledge around optimal radiotherapy dose, site location techniques, and interval between radiotherapy and PD-1 inhibitor treatments is lacking and must be explored in detail. In addition, insufficient is known about risk and harm associated with corresponding doses. Therefore while promising, we have a great deal to learn in devising appropriate PD-1 blockades combined with radiotherapy.

#### PD-1 blockades combined with traditional chemotherapy

A conventional strategy for enhancing the antitumor effect of immunotherapy is to combine with chemotherapy. Accumulating evidence indicates that chemotherapeutic drugs regulate the immune system while directly killing tumor cells by interfering with DNA synthesis and replication [86, 87]. Firstly, chemotherapy can induce immunogenic death of tumor cells because tumor-associated antigens are drained to the lymph nodes which hold the potential to increase the immune system’s ability to identify tumors. Cytotoxic agents (i.e., taxanes) block tumor proliferation and affect innate immune cell function within the tumor microenvironment [88]. Second, the use of chemotherapeutic drugs can activate the interferon pathway of tumors, increasing CD8^+^ T cell infiltration while providing a suitable microenvironment for anti-PD-1/PD-L1 therapy [89]. In addition, chemotherapy might actually inhibit the immune escape mechanism of tumor cells by inhibiting MDSCs via selective depletion of Tregs [90, 91].

In a mouse model of lung adenocarcinoma, Pfirschke et al. found that autochthonous tumors which lacked T cell infiltration and resisted current treatment options could be sensitized to host antitumor T cell immunity when chemotherapy drugs are applied [92]. However, this initial evidence has only moderate support in human populations. The KEYNOTE-021 study involving patients with advanced non-squamous NSCLC found a 56.7% ORR with pembrolizumab plus pemetrexed-Carboplatin (PC) compared with that of PC alone which was 30.2%. Also, as a first-line treatment, pembrolizumab combined with PC has the potential to reduce the risk of disease progression by 44% with prolonged longevity compared with the PC control group, 24 mPFS versus 9.3 month, respectively [93]. Due to fact that these participants were in the advanced stages of NSCLC, they had already received treatments which were not necessarily standardized. Logically, these treatments interact and are therefore likely to have added differential influences over the secondary treatment. Similar results have been found in patients with previously untreated metastatic non-squamous NSCLC without EGFR or ALK mutations. The results of the KEYNOTE-189 trial yielded a 69.2%, 12-month overall survival (OS) for the pembrolizumab combination group. While in the placebo combination group, the 12-month OS was only 49.4% [94].

**Fig. 2:**
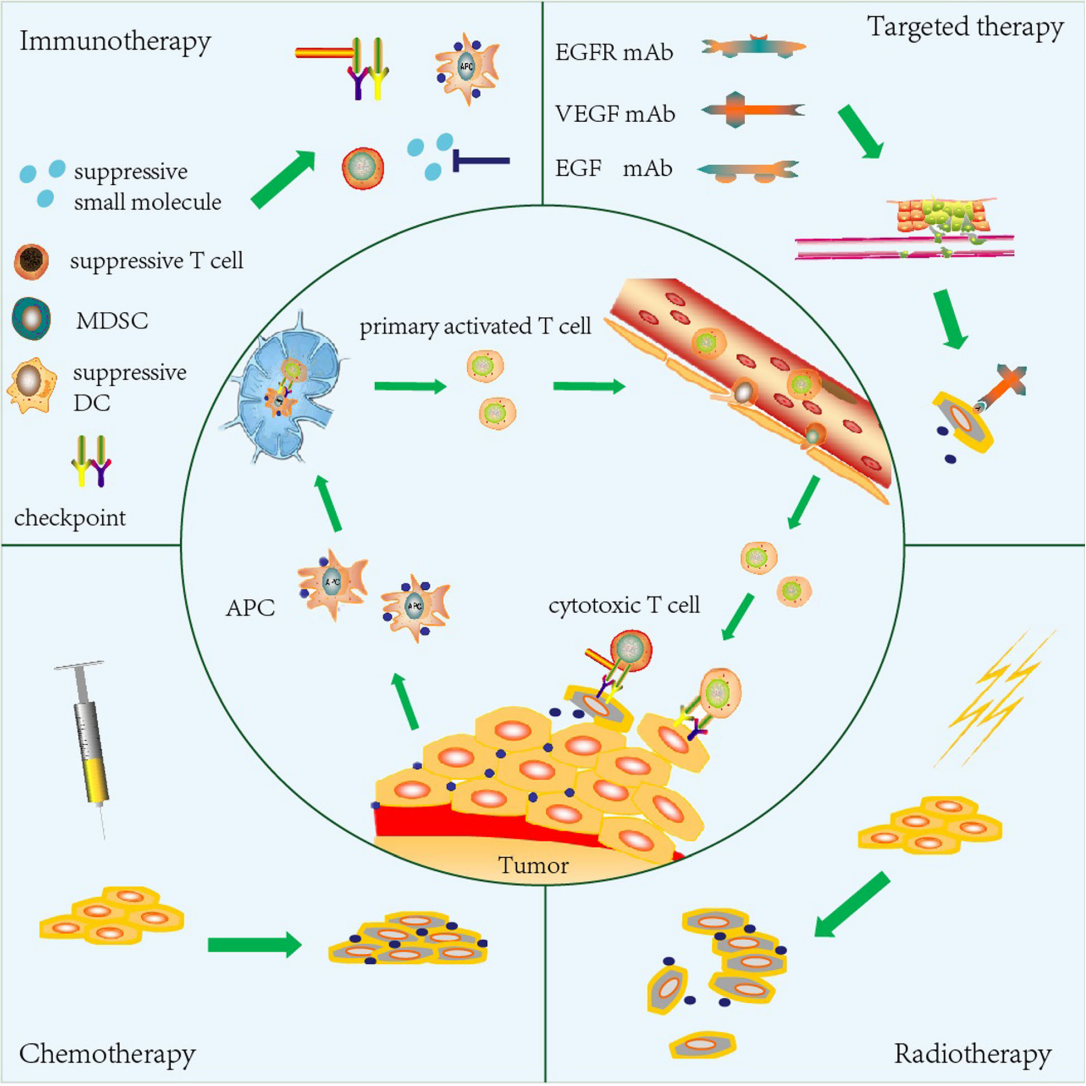
Combination strategy in tumor immune circulation. As described in the cancer-immunity cycle, there are three main stages involving the presentation of tumor cell antigen by the APC cells, primary activation of T cells in the lymph node, and migration of cytotoxic T cells from the vessel to kill the tumor cells. Several other types of antitumor therapy, such as radiotherapy, chemotherapy, another immunotherapy, and targeted therapy, can participate in the cancer-immunity cycle by destroying the tumor matrix, increasing antigen exposure, removing the immunosuppressive factors, promoting the infiltration of T cells, etc.

Again, many clinical studies are developing this evidence base around the efficacy of combined immunotherapies, though it remains necessary to monitor and report side effects. Furthermore, periodic administration of chemotherapeutic drugs may elicit a significant reduction in T lymphocytes which might weaken the immune effect of PD-1/PD-L1 blockades. It is therefore necessary to observe the CD4^+^/CD8^+^ status and adjust dosages according to individual responses. The variety of chemotherapeutic drugs is subtly different, and there is significant heterogeneity among tumor types. Hence, investigating this combination as opposed to ICPIs or chemotherapy alone must be conducted according to tumor classification and characteristics. The mechanism of combination immunotherapies has been summarized in Fig. 2.

## Current applications of combination immunotherapy in gastrointestinal tumors

Among the cluster of digestive tract tumors, histological differences are significant and are generally used to determine which approach to implement, especially for advanced tumors. For example, radiotherapy is efficacious in patients with esophageal cancer but not in patients with pancreatic cancer. Likewise, chemotherapy is the main stay for the treatment of patients with advanced gastric cancer but chemotherapeutic regimens are not generally administered for hepatocellular carcinoma. Therefore, combining superior interventions for digestive tract tumors with a single-drug immunotherapy may achieve enhanced immune expansion, despite the efficacy of PD-1/PD-L1 blockades varying substantially. Most studies are in the early phase clinical trials, although there are some which have progressed to phase III (Table 1). In this section, we systematically review officially published clinical studies for GI cancer sought through clinicaltrial.gov, PubMed, and in gray literature including conferences, such as ASCO and ESMO. Levels of efficacy will be critical discussed for several major digestive system tumors using relevant treatment indexes (i.e., OS, PFS, etc.).

**Table 1:** Ongoing phase 3 clinical trials of combined immunotherapy in gastrointestinal cancers

Tumor type	Phase/participants	Immune checkpoint inhibitors classification	Combination intervention	Status	ClinicalTrials.gov identifier
Unresectable, recurrent, locally advanced or metastatic gastric or gastroesophageal junction adenocarcinoma	3/371	PD-L1 inhibitors	Avelumab + BSC VS physician’s choice + BSC	Active, not recruiting	NCT02625623
Various advanced cancers	3/939	PD-1 and CTLA-4 inhibitors	Nivolumab + ipilimumab or nivolumab + fluorouracil + cisplatin VS fluorouracil + cisplatin	Recruiting	NCT03143153
Esophageal neoplasms	3/700	PD-1 inhibitors	Pembrolizumab + cisplatin and 5-fluorouracil (5-FU) VS placebo + cisplatin and 5-FU	Recruiting	NCT03189719
Esophageal carcinoma| esophagogastric junction carcinoma	3/720	PD-1 inhibitors	Pembrolizumab (MK-3475) VS Investigator’s Choice Standard Therapy	Active, not recruiting	NCT02564263
Gastric cancer	3/700	PD-1 inhibitors	Nivolumab + S-1 therapy or CapeOX therapy VS placebo+ S-1 therapy or CapeOX therapy	Recruiting	NCT03006705
Gastric cancer	2,3/680	PD-1 inhibitors	ONO-4538 + chemotherapy	Active, not recruiting	NCT02746796
Gastric cancer| gastroesophageal junction cancer	3/1649	PD-1 and CTLA-4 inhibitors	Nivolumab + ipilimumab or nivolumab + chemotherapy VS chemotherapy alone	Recruiting	NCT02872116
Gastric cancer| Gastroesophageal junction cancer	3/860	PD-1 inhibitors	Pembrolizumab (MK-3475) + chemotherapy VS placebo + chemotherapy	Recruiting	NCT03221426
Stomach neoplasms	3/780	PD-1 inhibitors	Pembrolizumab (MK-3475) + chemotherapy VS placebo + chemotherapy	Not yet recruiting	NCT03675737
Gastric neoplasms| gastroesophageal junction adenocarcinoma	3/732	PD-1 inhibitors	Pembrolizumab/Placebo + trastuzumab + Chemotherapy	Recruiting	NCT03615326
Gastric adenocarcinoma	3/764	PD-1 inhibitors	Pembrolizumab as monotherapy, or pembrolizumab + Cisplatin + 5-fluorouracil (5-FU) or capecitabine; placebo + cisplatin + 5-FU or capecitabine	Active, not recruiting	NCT02494583
Biliary tract neoplasms	3/390	PD-1 inhibitors	KN035 + gemcitabine + oxaliplatin VS gemcitabine + oxaliplatin	Recruiting	NCT03478488
Hepatocellular carcinoma	3/330	PD-1 inhibitors	Pembrolizumab (MK-3475) or placebo + Best supportive care	Recruiting	NCT03062358
Hepatocellular Carcinoma	3/480	PD-L1 inhibitors	Atezolizumab + bevacizumab VS sorafenib	Recruiting	NCT03434379
Hepatocellular carcinoma	3/1200	PD-L1 and CTLA-4 inhibitors	Durvalumab + tremelimumab	Recruiting	NCT03298451
Pancreatic cancer stage IV	2/40	PD-1 inhibitors	Nivolumab + cabiralizumab + gemcitabine VS gemcitabine	Not yet recruiting	NCT03697564
Colorectal cancer	3/363	PD-L1 inhibitors	Cobimetinib + atezolizumab and atezolizumab monotherapy VS regorafenib	Active, not recruiting	NCT02788279
Colorectal adenocarcinoma| mismatch repair deficiency	3/347	PD-L1 inhibitors	Atezolizumab, bevacizumab, Mfolfox6 VS bevacizumab, Mfolfox6 VS atezolizumab	Recruiting	NCT02997228
Colon Adenocarcinoma| DNA repair disorder	3/700	PD-L1 and CTLA-4 inhibitors	Combination chemotherapy with or without atezolizumab	Recruiting	NCT02912559
Colorectal cancer	3/180	PD-1 inhibitors	Nivolumab with standard of care therapy VS standard of care therapy for first-line treatment	Recruiting	NCT03414983

### Esophageal carcinoma

Moderate progress has been made in the diagnosis and treatment of esophageal cancer; however, the 5-year survival rate for patients with advanced esophageal cancer remains less than 15%. A PD-1 blockade is mainly administered for patients with advanced esophageal cancer, including patients showing first-line drug resistant esophageal cancer, or localized progression and advanced metastasis. In the USA, pembrolizumab has been approved for the treatment of patients with chemotherapy-refractory PD-L1-positive gastroesophageal junction cancers on the basis of clinical activity observed in the KEYNOTE-059 trial. This study found that 95 patients, representing 42.4%, experienced a reduction in measurable tumor size with a corresponding 11.6% ORR [95]. However, the KEYNOTE-180 study also found a 14% ORR for PD-1 blockages in esophageal squamous cell carcinoma patients compared with that of esophageal adenocarcinoma patients which was only 5%. This finding was lower than had been expected given the findings in the KEYNOTE-28 study where the ORRs of squamous cell carcinoma and adenocarcinoma were 29% and 40%, respectively [96, 97]. A subsequent phase III study, KEYNOTE-181 (NCT02564263), is currently looking to evaluate the activity of pembrolizumab versus a standard therapy in patients with metastatic esophageal carcinoma which progressed after receiving a first-line intervention. Preliminary outcomes suggest pembrolizumab is superior to chemotherapy for OS in PD-L1 with combined positive score ≥ 10 patients. The reported 12-month OS rate was 43% as opposed to 20%, and drug-related AEs associated with pembrolizumab were fewer than in the group which received chemotherapy alone (64% versus 86%).

ICPIs in esophageal cancer encourage optimism and combined with an immunotherapy may bring further benefit for those suffering esophageal cancer. Several clinical trials investigating PD-1 combined with radiotherapy have already been conducted with esophageal cancer patients. The CheckMate-032 study focused on double immunotherapeutic interventions for esophageal cancer and found a 24% ORR for nivolumab administered at 1 mg/kg plus ipilimumab administered at 3 mg/kg, with a corresponding PFS at 12 months of 17% (Table 2). This finding was twice that of the group which received nivolumab alone. However, the treatment-related grade 3 and 4 AEs in the combination group was 47%, whereas with the single agent intervention resulted in only 17%. The authors concluded that treatment with this ipilimumab combination significantly increased the incidence of side effects [98]. Finding an appropriate combination is clearly required, further necessitating the development of this evidence-base.

**Table 2:** Key trials of combination immunotherapy in esophagogastric cancers

Study/trial identifier	Tumor type	Phase	Participants	Combination intervention	Combination regimen type	Clinical endpoints	TRAEs	Reference
CheckMate-032; NCT01928394	Metastatic esophagogastric cancer	Ph-1/2	160	Nivolumab 3 mg/kg; nivolumab 1 mg/kg + ipilimumab 3 mg/kg; nivolumab 3 mg/kg + ipilimumab 1 mg/kg	PD-1 + CTLA-4	ORR: 12%, 24%, 8%; PFS rate at 12 months: 8%, 17%, 10%; OS rate at 12 months: 39%, 35%, 24%	Grade 3/4: 17%, 47%, 27%	2018 JCO; [98]
KEYNOTE-059 cohort 2; NCT02335411	Advanced gastric cancer	Ph-2	25	Pembrolizumab + 5-fluorouracil + cisplatin	PD-1 + Chemo	ORR: 60%; mPFS: 6.6 months; mOS: 13.8 months	Grade 3/4: 76%	2017 ASCO; [99]
NCT02999295	Advanced gastric adenocarcinoma	Ph-1/2	46	Ramucirumab + nivolumab	PD-1 + Target	PR: 22%; DCR: 59%	Any grade: 87%; Grade 3/4: 13%	2018 ASCO; [100]
NCT02013154	Advanced gastroesophageal cancer	Ph-1	13	Dickkopf-1 + pembrolizumab	PD-1 + Molecules	PR: 1 pt/9 pts; SD: 5 pts/9 pts; DCR at 6 weeks: 75%	Grade ≥ 3: 4 pts./9 pts	2018 ESMO; [101]
Attraction-04; NCT02746796	Unresectable advanced or recurrent G/GEJ cancer	Ph-2/3	40	Nivolumab + oxaliplatin + S-1,or capecitabine; placebo + oxaliplatin + S-1, or capecitabine	PD-1 + Chemo	ORR: 68.4%; DCR: 86.8%;	Grade 3/4: 57.5%	2018 ESMO; [102]
NCT02689284	Advanced HER2+ gastric adenocarcinoma	Ph-1/2	60	Margetuximab + Pembrolizumab	PD-1 + Target	ORR: 21.6%; DCR: 55%; mOS: 15.6 months	Grade ≥ 3: 16.7%	2018 ESMO; [103]
NCT02864381	Unresectable or recurrent gastric or gastroesophageal junction adenocarcinoma	Ph-2	144	Andecaliximab 800 mg + nivolumab 3 mg/kg, or nivolumab 3 mg/kg alone	PD-1 + Molecules	ORR: ADX + nivo, 11.1%; nivo alone, 6.9%; mPFS: ADX + nivo, 1.8 months; nivo alone, 1.9 months; mOS: ADX + nivo, 7.2 months; nivo alone, 5.9 months	AEs leading to treatment discontinuation: ADX + NIVO, 1 pt.; nivo, 1 pt	2019 ASCO; [104]
NCT02954536	HER2-positive metastatic esophagogastric adenocarcinoma	Ph-2	24	Pembrolizumab + chemotherapy/trastuzumab	PD-1 + Chemo/Target	ORR: 83%; mPFS: 11.4 months	Gr 2 fatigue (35%), Gr 2/3 nausea (35%), Gr 2 diarrhea (26%), Gr2 AST/ALT elevation (16%), Gr2 neutropenia (16%)	2019 ASCO; [105]
NCT02689284	Advanced HER2+ (IHC3+) gastric carcinoma	Ph-1/2	66	Margetuximab 15 mg/kg + pembrolizumab 200 mg	PD-1 + Target	ORR: 41.4%; DCR: 72.4%; mPFS: 5.5 months	Grade ≥ 3: 18.2%	2019 ASCO; [106]
NivoRam study; NCT02999295	Advanced gastric adenocarcinoma	Ph-1/2	46	Nivolumab 3 mg/kg, Q2W + ramucirumab 8 mg/kg, Q2W	PD-1 + Target	ORR: 26.7%; PFS rate at 6 months: 37.4%; mPFS: 2.9 months; mOS: 9.0 months	Grade 3/4: hypertension, diarrhea, perforation at jejunum, hemorrhage, colitis, pancreatitis, liver dysfunction, cholangitis, hematoma, neutropenia and proteinuria	2019 ASCO; [107]

Based on current findings, a further phase III studies (NCT02872116) was designed to evaluate double immunotherapy as an early line therapy for esophagogastric cancers, and is presently under way. For the PD-1 and chemotherapy combination, the NCT03189719 trial is ongoing to evaluate the efficacy and safety of pembrolizumab plus cisplatin and 5-fluorouracil (5-FU) chemotherapy versus placebo plus cisplatin and 5-FU chemotherapy as a first-line treatment in participants with locally advanced or metastatic esophageal carcinoma. In fact, the majority of trials in this field are still in exploratory phases involving a variety of combinations. While results are pending, current knowledge provides some optimism and the results are eagerly anticipated.

### Gastric carcinoma

The Cancer Genome Atlas (TCGA) divides gastric cancer into an Epstein-Barr virus (EBV) positive subtype, a microsatellite instability (MSI) subtype, a genomically stable (GS) subtype, and the chromosomal instability (CIN) subtype, according to histologically based integrative genomics [108]. Among the four types of gastric cancer, the high-frequency MSI (MSI-H) subtype appears to respond favorably [109]. The results of the ATTRACTION-02 phase III study focusing on heavily pretreated patients with advanced gastric or gastroesophageal junction cancer found OS rates in nivolumab compared with placebo were 27.3% and 11.6% at 12 months, and then 10.6% and 3.2% at 24 months, respectively. However, the nivolumab ORR was only in 11% of 268 patients which was considered a relatively low response rate [110].

Comparatively, the KEYNOTE-061 trial which focused on pembrolizumab with paclitaxel in patients with advanced gastric cancer whom had developed resistance after platinum and fluoropyrimidine treatment found that pembrolizumab did not significantly improve OS compared to paclitaxel, with an 9.1 month mOS versus 8.3 months [111]. Unsatisfactory immune monotherapies in gastric cancer make combined therapy especially enticing. Although, most of the combination strategies being investigated in gastric cancer are in the preclinical or early clinical research stage, few have entered the phase III stage [112]. For example, the CheckMate-649 is further assessing the difference in survival between nivolumab plus ipilimumab and chemotherapy although results are pending.

In the KEYNOTE-059 cohort 2 study, the ORR and DCR of 25 patients with advanced gastric or gastroesophageal adenocarcinoma were 60% and 80%, and the median PFS and OS were 6.6 and 13.8 months, respectively. Subgroup analysis highlighted a 69% ORR in PD-L1-positive patients and 38% in PD-L1-negative patients [99] (Table 2). This small sample study suggests that chemotherapy combined with anti-PD-1 has potential in gastric or gastroesophageal conjunctive adenocarcinoma, although confirmatory findings are required. In a related follow up, an investigation of the efficacy of chemotherapy combined with PD-1 blockades, KEYNOTE-062, is in progress to assess this combination as a first-line therapy for advanced gastric or gastroesophageal junction adenocarcinoma.

The preliminary results of a phase I/II study of ramucirumab plus nivolumab in patients with previously treated advanced gastric adenocarcinoma found a partial response was obtained in ten patients, representing a 22% of the study population with a DCR of 59% [100]. In addition, a phase I study (NCT02443324), which assessed the efficacy of pembrolizumab in combination with ramucirumab, found a 50% DCR and PD-L1-positive patients appear to have significantly benefited [111]. Combination immunotherapies in esophageal and gastric cancer have achieved a preliminary advantage, and sequencing combination therapies is also moving forward.

### Hepatobiliary carcinoma

Presently, targeted drugs, such as sorafenib, lenvatinib, and regorafenib, are the primary treatments for advanced hepatocellular carcinomas (HCC). Recent results have indicated the potential of PD-1/PD-L1 blockades for the treatment of advanced HCC. In the CheckMate-040 study, the overall ORR of the patients administered with nivolumab was 14–23%. Subgroup analysis suggested that the DCR in patients without sorafenib was 54% with an OS of 28.6 months. In patients treated with sorafenib, the ORR was 55%, suggesting that there may be only a fractional benefit, although this group had a prolonged 15.6 month OS [113]. In addition, liver toxicity of PD-1/PD-L1 blockades was lower than that of conventional drugs. As a result in 2017, nivolumab was approved by the FDA as a second-line treatment for HCC. Preliminary results from the KEYNOTE-224 study are similar to those of CheckMate-040, the ORR, and DCR in patients with advanced HCC whom had previously been treated with sorafenib was 17% and 61%, respectively [114]. In view of the aforementioned findings, the phase III CheckMate-459 trial which will compare nivolumab with sorafenib as first-line treatments for advanced HCC with overall survival as the primary endpoint is much needed [115].

PD-1 inhibitor monotherapies appear to be well tolerated with relatively consistent efficacy in liver cancer patients. For example, the retrospective study of CheckMate-040 trial found a 50% ORR in 14 patients whom had received nivolumab combined with local-regional treatment with three CRs (11%) and five PRs (18%) [116]. To further increase the antitumor response, a preliminary study of lenvatinib plus pembrolizumab in patients with unresectable HCC resulted in encouraging antitumor activity and tolerance with 46% ORR (Table 3). The most common AEs were decreased appetite and hypertension without new safety signals [117].

**Table 3:** Key trials of combination immunotherapy in hepatocellular, biliary tract, and pancreatic cancers

Study/trial identifier	Tumor type	Phase	Participants	Combination intervention	Combination regimen type	Clinical endpoints	TRAEs	Reference
CheckMate-040 retrospectively evaluate;	Advanced HCC		28	Nivolumab + local-regional treatment	PD-1 + LR	ORR: 50%; SD: 21%; mOS: 13.6 months	Grade 3: 7%	2018 ASCO; [116]
NCT03006926	Unresectable HCC;	Ph-1b	13	Lenvatinib + pembrolizumab	PD-1 + Target	ORR: 46%; SD: 46%;	Any grade: 94%; decreased appetite: 56%; hypertension: 56%	2018 ASCO; [117]
NCT02715531	Unresectable or metastatic HCC	Ph-1b	68	Atezolizumab + bevacizumab	PD-L1 + Target	ORR: 34%; PFS rate at 6 months: 71%	Any grade: 72%; Grade 3/4: 25%	2018 ESMO; [118]
Study-022; NCT02519348	Advanced HCC	Ph-1/2	40	Durvalumab + tremelimumab	PD-L1 + CTLA-4	ORR: 18%; DCR: 57.5%	Any grade: 72%; Grade 1–3: 20%	2017 ASCO; [119]
JapicCTI-153098	Biliary tract cancer	Ph-1	30	Nivolumab 240 mg, 2-week intervals + cisplatin-gemcitabine	PD-1 + Chemo	ORR: 36.7%; mPFS: 4.2 months; mOS: 15.4 months	Malaise (8/30, 27%) and decreased appetite (7/30, 23%)	2019 ASCO; [120]
2018 ASCO Poster	Advanced intrahepatic cholangiocarcinoma		14	Lenvatinib + pembrolizumab or nivolumab	PD-1 + Targeted	ORR: 21.4%; DCR: 92.9%; mPFS:5.9 months	Grade 3: 14%	2018 ASCO; [121]
NCT01938612	Biliary tract cancer	Ph-1	65	Durvalumab 20 mg/kg + tremelimumab 1.0 mg/kg, q4w; durvalumab monotherapy	PD-L1 + CTLA-4	DCR: D, 16.7%; D + T, 32.2%; mPFS: D, 9.7 months, D + T, 8.5 months; mOS: D, 8.1 months; D + T, 10.1 months	Any grade: D, 64%; D + T, 82%; Grade ≥ 3: D, 19%; D + T, 23%; D + T: a death due to drug-induced liver injury	2019 ASCO; [122]
NCT02821754	Advanced HCC; advanced BTC	Ph-2	22	Monthly tremelimumab 75 mg + durvalumab 1500 mg for 4 doses followed by monthly durvalumab 1500 mg monotherapy	PD-L1 + CTLA-4	ORR: HCC, 20%; BTC, 0%; DCR: HCC, 60%; BTC, 41.7%; mPFS: HCC, 7.8 months, nivo alone, 3.1 months; mOS: HCC, 15.9 months; BTC, 5.45 months	Grade 3/4: lymphocytopenia, hyponatremia, bullous dermatitis, maculopapular rash	2019 ASCO; [123]
KEYNOTE-202; NCT02826486	Metastatic pancreatic adenocarcinoma	Ph-2a	37	BL-8040 + pembrolizumab	PD-1 + Molecules	PR: 3.4%; DCR: 34.5%; mOS: 3.4 months; OS rate at 6 months: 34.9%		2018 ESMO; [124]
NCT02309177	Advanced pancreatic cancer	Ph-1	50	Nab-paclitaxel 125 mg/m^2^ + gemcitabine 1000 mg/m^2^ on day 1, 8, and 15 + nivolumab 3 mg/kg on d 1 and 15 of each 28-day cycle	PD-1 + Chemo	ORR: 18%; DCR:64%; mPFS: 5.5 months; mOS: 9.9 months	Grade 3/4: 96%; Grade 5: 1 pts	2019 ASCO; [125]
NCT02311361	Advanced pancreatic adenocarcinoma	Ph-1/2	51	Cohort 1: Durvalumab + SBRT; Cohort 2: SBRT followed by durvalumab; Cohort 3: Durvalumab + Tremelimumab + SBRT; Cohort 4: SBRT followed by Durvalumab + Tremelimumab	PD-L1 + CTLA-4 + RT; PD-L1 + RT	PR: cohort 1, 1 pt.; cohort 4, 2 pts.; mPFS and mOS: cohort 1, 1.7 months, 3.4 months; cohort 2, 2.6 months, 9.1 months; cohort 3, 1.6 months and 3.0 months; cohort 4, 3.2 months, 6.4 months	The most commonly TRAEs were lymphopenia. Grade 3–4: lymphopenia and anemia	2019 ASCO; [126]

The FDA recommends atezolizumab combined with bevacizumab as a first-line therapeutic regimen for patients with advanced HCC based on a phase 1b study (NCT02715531). The findings of this study highlight a 34% ORR associated with atezolizumab combined with bevacizumab among 68 patients assessed [118], although this was a relatively small study. The recent phase III IMbrave150 trial is based upon these findings and will evaluate the efficacy and safety of this combination compared to sorafenib in participants with locally advanced or metastatic HCC who have received no prior systemic treatment [127]. Combining PD-1 blockade and CTLA-4 blockade for advanced HCC may also prove beneficial and early data from NCT02519348 suggests relative safety with an 18% ORR [113] and the upgraded study is currently recruiting. In addition, several clinical trials of PD-1/PD-L1 blockades combined with other types of antitumor therapy are also under way.

Related basic medical research by Nakamura et al. divided biliary tract cancers (BTC) into four molecular subgroups based upon prognostic gene profiles and found that classification correlates with patient prognosis. Among subtypes with the worst prognosis, the expression of immune checkpoint-related molecules, including PD-L1, was upregulated more than in any other subgroups, which again suggests immune checkpoint inhibitors may yield a favorable response [128]. In addition, emerging data suggests MMR or MSI-H mutation tumors have a much higher response rate to PD-1/L1 inhibitors, and in cholangiocarcinoma, MSI-H accounting for 5% of gallbladder cancers (GBC), 5–13% of extrahepatic cholangiocarcinoma (ECC), and 10% of intrahepatic cholangiocarcinoma (ICC) [109]. Phage 1b KEYNOTE-028 trail assessed the safety and activity of pembrolizumab monotherapy among advanced solid tumors with PD-L1 expression ≥ 1%, and the cholangiocarcinoma cohort suggested that of 24 patients who met the evaluation criteria ORR was 17% [129].

Sequencing exons and transcriptomes has revealed heterogeneous molecular changes among cholangiocarcinoma, and the selection of an immunotherapy combined with a targeted therapy may provide answers where other avenues may not. One small sample study found after treatment with PD-1 blockades combined with lenvatinib, 3:14 patients had a 21.4% ORR and a 93% DCR. Interestingly, this study using 450-gene next generation sequencing (NGS) panel in seven patients to detect all classes of genetic status discovered that having a high TMB might be used to indicate preferential treatment [121] (Table 3). The standard first-line chemotherapy for advanced BTC is gemcitabine plus cisplatin; however, there is no standardized second-line intervention. This is because evidence is lacking to guide specialists. PD-1/L1 blockades combined with a standard chemotherapy is frequently administered as a second-line therapy, although there appears to be an element of trial and error adjustment. Currently, several clinical trials are under way, including one investigating a guadecitabine and durvalumab combination (NCT03257761) and another pembrolizumab and FOLFOX (NCT02268825) (Table 3). The findings of these studies may provide support for clinicians seeking the most effective option where first-line treatments have failed.

Another interesting research avenue which has emerged is around the impact of standards of care (SoC). Currently under way, a phase III clinical study is exploring this in more detail, focusing on the efficacy of PD-1 blockade combined with SoC compared with SoC alone for the treatment of previously untreated locally advanced or metastatic BTC. The primary hypothesis of the study is that participants will have a longer OS when treated with combined therapy than when treated with SoC alone, although this study may also provide insight into the interactions between SoC and PD-1 blockades which is also needed.

### Pancreatic carcinoma

Previously presented evidence suggests that immunotherapy combined with PD-1/PD-L1 blockades may deliver favorable outcomes with durable responses for various types of cancer; however, pancreatic carcinomas remain refractory. Except for MSI-positive pancreatic cancers which accounts for approximately 1.2%, the efficacy of PD-1/PD-L1 blockades alone are unsatisfactory for most pancreatic cancers. Unfortunately, more than 10% of patients develop grade 3 and 4 AEs, which is likely to be at least partly be due to the unique microenvironments (TME) in the pancreas [130]. Pancreatic tumor tissues are characterized by excessive cancer-associated fibroblasts (CAFs), dense connective tissue, low vascular density, and insensitivity to ischemia and hypoxia. In addition, immunosuppressive immune cells, such as M2 macrophages, are found in tumor tissues which inhibit immune killer cells from effectively entering through the tumor matrix [131]. Potentially, combined immunotherapies could provide a solution to these problems by bolstering the immune response to pancreatic tumor development.

Presently, gemcitabine, albumin paclitaxel, and a monoclonal CD40 antibody combined with nivolumab are frequently used as pancreatic cancer interventions. These interventions act by destroying tumor matrices and by exposing more antigens, which promote lymphocyte infiltration. Cabiralizumab (FPA008) is an anti-CSF-1R antibody which can cause the depletion of tumor-associated macrophages (TAMs) which may provide additional benefit. As such, one recent study (NCT02526017) was designed specifically to evaluate the safety, tolerability, as well as the clinical benefit of cabiralizumab in combination with nivolumab in patients with selected advanced cancers, including pancreatic cancer. The study revealed lasting clinical benefit for five patients with advanced pancreatic cancer who were insensitive to a previously administered single-drug immunotherapy, including three patients with microsatellite stability (MSS). However, the sample size of the study was small (*n* = 33), therefore these results ought be verified under stricter conditions, including a larger sample size based on a pre-trial calculation using best available evidence, and with an appropriate control group. Importantly, it is necessary to conduct this research focusing on those suffering pancreatic cancer specifically because of the refractory nature of this condition but also to explore therapeutic effects across stages.

A phase II clinical trial (NCT03336216) currently under way is focusing on the efficacy of cabiralizumab and nivolumab combined with or without chemotherapy specifically for the treatment of *advanced* pancreatic cancer. Chemotherapy in this particular trial includes paclitaxel, gemcitabine, irinotecan, or FOLFIRINOX. The researchers have proposed to recruit 160 patients which is substantially larger than previously mentioned NCT02526017 study, and to use PFS as the primary clinical endpoint. The potential benefit of PD-1/PD-L1 blockades combined with other therapeutic approaches has resulted in a number of trials focusing on resectable pancreatic cancer, broad line resectable pancreatic cancer, and advanced pancreatic cancer. Most of the trials being designed are again preclinical studies or early phase clinical research but hopefully findings from the aforementioned studies will develop this evidence base and drive higher level clinical research.

### Colorectal carcinoma

The KEYNOTE-028 trial which involved a cohort of people with existing colon and rectum carcinomas found only a 4% ORR for pembrolizumab monotherapy after screening out patients with PD-L1 > 1% (*n* = 1), and there was no significant improvement when compared with that of unscreened patients [132]. DMMR/MSI-H-type mCRC accounts for 4% of mCRC overall, although this is insensitive to traditional chemotherapy and generally has a poor prognosis. However, many neoantigens increase dMMR patients’ sensitivity to PD-1/PD-L1 blockade therapy. Therefore, nivolumab has been approved for patients with metastatic DNA mismatch repair-deficient colorectal cancer based on the Checkmate 142 study suggesting 23 of 74 patients achieved objective response and 68.9% of patients had disease control for ≥ 12 weeks [133].

Nevertheless, the colorectal cancer group of phase II clinical trials evaluating the clinical activity of pembrolizumab in patients with progressive metastatic carcinoma has shown that the ORR and DCR of patients with mismatch repair-deficient (dMMR) within 20 weeks were 40 and 90%, respectively. For the mismatch repair-proficient (pMMR) group, these values were 0 and 11%, respectively which suggests that mismatch repair status may be used as efficient indicators of PD-1 antibodies, although further research is needed for clarification [109]. One phase 3 clinical trial (NCT02563002) has been designed to investigate these issues and will compare PFS and OS between dMMR/MSI-H patients administered single-drug PD-1 inhibitor therapy and dMMR/MSI-H patients administered standard chemotherapy.

Concerning double immunotherapy in dMMR/MSI-H mCRC, results for the nivolumab plus ipilimumab cohort of CheckMate-142 study found at the median follow-up (13.4 months) a 55% ORR with corresponding PFS and OS rates at 12 months of 76% and 87%, respectively [134] (Table 4). Therefore, indirect comparisons suggest that combination therapies provide improved efficacy relative to anti-PD-1 monotherapy (ORR 31%) and has a favorable benefit-risk profile. Importantly, the study also suggests that there is no relationship between efficacy and the expression of PD-L1 in MSI-H patients.

**Table 4:** Key trials of combination immunotherapy in colorectal cancers

Study/trial identifier	Tumor type	Phase	Participants	Combination intervention	Combination regimen type	Clinical endpoints	TRAEs	Reference
CheckMate-142; NCT02060188	DNA mismatch repair-deficient/microsatellite instability-high metastatic colorectal cancer	Ph-2	119	Nivolumab 3 mg/kg + ipilimumab 1 mg/kg q3w, followed by nivolumab 3 mg/kg once q2w	PD-1 + CTLA-4	ORR: 55%; DCR: 80%; PFS rate at 12 months: 71%; OS rate at 12 months: 85%	Grade 3/4: 32%	2018 JCO; [134]
NCT01633970	Metastatic colorectal cancer	Ph-1	44	Arm A, MPDL3280A (anti-PDL1) + bevacizumab; Arm B, MPDL3280A + bevacizumab + FOLFOX	PD-L1 + Target; PD-1 + Target + Chemo	ORR: Arm A, 8%; Arm B, 36%;	Grade 3/4: Arm A, 64%; Arm B, 73%	2017 ASCO; [135]
NCT01988896	Metastatic colorectal cancer	Ph-1b	84	Atezolizumab + cobimetinib	PD-L1 + Target	ORR: 8%; DCR: 31%; OS rate at 12 months: 43%; mOS: 9.8 months; PFS rate at 6 months: 18%	Any grade: 96%; Grade 3/4: 32%	2018 ASCO; [136]
NCT02375672	Colorectal cancer irrespective of MMR status	Ph-2	40	Pembrolizumab + mFOLFOX6	PD-1 + Chemo	ORR: 53%; DCR at 8 weeks: 100%	Grade 3/4: 36.7%	2017 ASCO; [135]
NCT02437071	Mismatch repair proficient (pMMR) metastatic colorectal cancer	Ph-2	26	Pembrolizumab + radiotherapy; pembrolizumab + ablation	PD-1 + RT; PD-1 + LR	ORR: RT cohort, 9%; LR cohort, no responses	Grade 1/2: 73%	2017 ASCO; [137]
NCT02981524	Mismatch repair–proficient (MMR-p) advanced colorectal cancer	Ph-2	17	Pembrolizumab + CyGVAX colon vaccine + cyclophosphamide	PD-L1 + vaccine + chemo	ORR: 18%; mPFS: 2.7 months; mOS: 7.0 months	Two patients (12%) had grade 3/4 adverse events that were attributed to study therapy	2019 ASCO; [138]
NCT02870920	Advanced refractory colorectal carcinoma, not selected for dMMR	Ph-2	180	Durvalumab 1500 mg D1 q 28 days + tremelimumab 75 mg D1 for first 4 cycles vs BSC	PD-1 + CTLA-4	DCR: D + T, 22.7%; BSC, 6.6%; mOS: D + T, 6.6 months, BSC, 4.1 months	Grade 3/4: abdominal pain, fatigue, lymphocytosis and eosinophilia were significantly higher in D + T	2019 ASCO; [139]

As mentioned previously, PD-1 inhibitor monotherapy has little effect in patients with microsatellite stable colorectal cancer. Indeed, many factors may influence the efficacy of PD-1/PD-L1 blockade in patients with colorectal cancer, including gene mutations, the immune microenvironment, and a patient’s genetic inheritance. In unscreened patients with advanced colorectal cancer, a small sample study at the 24-week follow-up found 53% ORR for PD-1 blockade combined with chemotherapy. Although, it remains unclear how effective chemotherapy alone will be for this group of patients due to the lack of rigorous experimental design, and the proportion of people (36.7%) suffering associated severe side effects associated [135].

MEK inhibition upregulates tumor major histocompatibility complex-I expression, promoting intra-tumoral T cell accumulation while improving anti-PD-L1 responses [140]. For patients with MSS colorectal cancer, recent studies have found that cobimetinib (MEK1/2 inhibitor) combined with PD-L1 blockades results in a DCR of 31%, and 43% of patients survive for more than 12 months [136]. As a result, a phase III clinical trial (NCT02788279) was designed to evaluate atezolizumab in combination with cobimetinib versus atezolizumab or regorafenib monotherapies and the findings are eagerly anticipated.

An increasing number of clinical trials are currently under development and ongoing which provides some optimism. However, these combinations face a number of problems, such as the need for more comprehensive gene sequencing and the difficulty of accurately and rigorously classifying colorectal cancer patients to predict treatment efficacy. In addition, the use of the same treatment regimen for different patients may not improve prognoses due to significant differences among individual patients which suggest the need for personalized cancer care. However, in order for this to become a reality, studies need to be scaled up and studies ought to be designed to incorporate the subtle differences between participants, which one could argue is not the current state of play.

## Conclusions and perspectives

The advantages of combined immunotherapy based on PD-1/PD-L1 blockades for various tumors appear to be the logical next step. Although, there are a great number of unknowns, including dose/response, safety, tolerability, durability, and indeed efficacy. How these new treatment options will be placed within the existing treatment framework is a concern. Researchers are endeavoring to answer these questions through rigorous clinical trials focusing on specific types of tumors and within specific populations at various stages of these diseases. Studies have found an increase in the proportion of immune-related adverse events after receiving combination therapy compared to monotherapies. Although, these generally include diarrhea, fatigue, and hypothyroidism, which are within a tolerable range and manageable [17].

The increasing the number of combination studies has highlighted beneficial antitumor effects in early clinical stages. However, results from several clinical trials found no enhanced benefit for the patients with advanced cancers. Moreover, administering combination immunotherapies has been found to increase treatment toxicity. In patients who received radiotherapy prior to treatment with PD-1 blockades, research has revealed that immune inflammation frequently and naturally recurs at the original site of irradiation. Therefore, as many of the current combined immunotherapeutic methods remain experimental, developing this evidence base is absolutely necessary.

Understanding the underlying mechanisms of each therapeutic combination as well as the subtleties of individual responses is required to avoid combination schemes which do harm. Ironically, combination immunotherapeutic models pose similar questions to traditional treatment: What is the ideal patient population for which combination? Is the required combination therapy sequential or concurrent? What timing and adjustment criteria can be used for continuous and combined interventions? What is the related safety and toxicity of each combination? All of these questions require a sophisticated evidence-base developed through mature theoretical foundations and basic medical research. Once small sample studies have been conducted, larger studies ought to be commenced as is currently occurring. However, at present, it would appear as though we are trying to improve outcomes by combining a possible best available treatment with a potential catalyst or less subtly, simply seeking compatible combinations. We must not overlook the fact that this is essentially combining an average of averages with yet another. More specific research is required with more comprehensive data collection if we are to treat individuals with more precision and sensitivity as is required for gastrointestinal malignancies. Further research should focus on markers as these may provide measurable trajectories to accurately predict the benefit of combination therapies.

## Abbreviations

5-FU: 5-Fluorouracil
AEs: Adverse events
APCs: Antigen-presenting cells
ASCO: American Society of Clinical Oncology
B2M: Beta-2-microglobulin
B7-H1: B7 homolog 1
BTC: Biliary tract cancer
CAFs: Cancer-associated fibroblasts
CCL5: Cell chemokine ligand 5
CIN: Chromosomal instability
CRC: Colorectal cancer
CTLA4: Cytotoxic T lymphocyte antigen-4
CXCL10: C-X-C motif chemokine 10
CXCR2: C-X-C Motif chemokine Receptor 2
DCR: Disease control rate
dMMR: Mismatch repair deficient
DOR: Duration of response
EBV: Epstein-Barr virus
EC: Esophagus cancer
ECC: Extrahepatic cholangiocarcinoma
ECCO: European Cancer Congress
EGFR: Epidermal growth factor receptor
FDA: Food and Drug Administration
GBC: Gallbladder cancer
GC: Gastric cancer
GEC: Gastroesophageal junction cancer
GI: Gastrointestinal
GITR: Glucocorticoid-induced TNFR family related gene
GS: Genomically stable
HCC: Hepatocellular carcinoma
HDAC: Histone deacetylase
HLA: Human leukocyte antigen
ICC: Intrahepatic cholangiocarcinoma
ICPIs: Immune checkpoint inhibitors
IDO: Indoleamine 2,3-dioxygenase
INV: Investigator
irAEs: Immune-related adverse events
JAK: Janus kinases
LAG-3: Lymphocyte-activation gene 3
MANAs: Mutation-associated neoantigens
McAbs: Monoclonal antibodies
MDM2/4: Murine double minute 2/4
MDSCs: Myeloid-derived suppressor cells
MEK: MAP kinse-ERK kinase
MHC: Major histocompatibility complex
mOS: Median overall survival
MSI-H: Microsatellite instability-high
MSS: Microsatellite stability
NSCLC: Non-small cell lung cancer
ORR: Objective response rate
OS: Overall survival
P13K: Phosphoinositide 3-kinase
PC: Pancreatic cancer
PD: Progressive disease
PD-1: Programmed cell death-1
PD-L1: Programmed cell death ligand-1
PD-L2: Programmed cell death ligand-2
PFS: Progression-free survival
pMMR: mismatch repair proficient
PR: Partial response
RCTs: Randomized clinical trials
SCCA: Squamous cell carcinoma of the anal canal
SCCs: Squamous cell carcinomas
SD: Stable disease
TAMs: Tumor-associated macrophages
TCGA: The Cancer Genome Atlas
TCR: T cell receptor
TILs: Tumor infiltrating lymphocytes
TIM-3: T cell immunoglobulin mucin-3
TMB: Tumor mutational burden
TME: Tumor microenvironment
TNFRSF4: Tumor necrosis factor receptor super family member 4
TRAEs: Treatment-related adverse events
Tregs: T regulatory cells
TR-TEAEs: Treatment-related treatment-emergent adverse events
VEGF: Vascular endothelial growth factor
